# Novel Paramyxoviruses in Free-Ranging European Bats

**DOI:** 10.1371/journal.pone.0038688

**Published:** 2012-06-21

**Authors:** Andreas Kurth, Claudia Kohl, Annika Brinkmann, Arnt Ebinger, Jennifer A. Harper, Lin-Fa Wang, Kristin Mühldorfer, Gudrun Wibbelt

**Affiliations:** 1 Robert Koch Institute, Centre for Biological Security, Berlin, Germany; 2 CSIRO Livestock Industries, Australian Animal Health Laboratory, Victoria, Australia; 3 Leibniz Institute for Zoo and Wildlife Research, Berlin, Germany; Institut Pasteur, France

## Abstract

The zoonotic potential of paramyxoviruses is particularly demonstrated by their broad host range like the highly pathogenic Hendra and Nipah viruses originating from bats. But while so far all bat-borne paramyxoviruses have been identified in fruit bats across Africa, Australia, South America, and Asia, we describe the detection and characterization of the first paramyxoviruses in free-ranging European bats. Moreover, we examined the possible impact of paramyxovirus infection on individual animals by comparing histo-pathological findings and virological results. Organs from deceased insectivorous bats of various species were sampled in Germany and tested for paramyxovirus RNA in parallel to a histo-pathological examination. Nucleic acids of three novel paramyxoviruses were detected, two viruses in phylogenetic relationship to the recently proposed genus Jeilongvirus and one closely related to the genus Rubulavirus. Two infected animals revealed subclinical pathological changes within their kidneys, suggestive of a similar pathogenesis as the one described in fruit bats experimentally infected with Hendra virus.

Our findings indicate the presence of bat-born paramyxoviruses in geographic areas free of fruit bat species and therefore emphasize a possible virus–host co-evolution in European bats. Since these novel viruses are related to the very distinct genera Rubulavirus and Jeilongvirus, a similarly broad genetic diversity among paramyxoviruses in other *Microchiroptera* compared to *Megachiroptera* can be assumed. Given that the infected bats were either found in close proximity to heavily populated human habitation or areas of intensive agricultural use, a potential risk of the emergence of zoonotic paramyxoviruses in Europe needs to be considered.

## Introduction

Members of the virus family *Paramyxoviridae* are divided into two subfamilies, *Paramyxovirinae* and *Pneumovirinae*, comprising a vast variety of animal- and human-pathogenic viruses [1]. Within the subfamily *Paramyxovirinae*, five genera have been classified, Respiro-, Morbilli-, Rubula-, Avula-, and Henipavirus, as well as a fast-growing group of unclassified viruses. The increased molecular characterization of recently isolated paramyxoviruses indicates a much greater genetic diversity within the subfamily *Paramyxovirinae* than previously assumed. Furthermore, the detection of highly human-pathogenic paramyxoviruses has also influenced the attention drawn to paramyxovirus research and to the isolation of further novel paramyxoviruses from hosts that are suggested as likely species to transmit newly emerging viruses. Bats are among this highly suspected group of animals [2]. They belong to the most successful and diverse mammals on earth and comprise approximately 1,200 chiropteran species distributed worldwide. In the last two decades important zoonotic viruses including Ebola, Marburg, and SARS virus, but also paramyxoviruses such as Hendra and Nipah virus have been identified in various *Pteropus* spp. (flying foxes) fruit bats [3]–[11]. For Menangle virus, another paramyxovirus isolated from fruit bats, less pathogenic courses of disease in humans have been described [12]. For other bat paramyxoviruses isolated, infections in humans have yet to be associated, e.g. Tioman virus from flying fox [13], bat parainfluenza virus from flying fox [14], Tuhoko virus from flying fox [6], Mapuera virus from non-pteropid fruit bat [15], and Henipa-like viruses also from non-pteropid fruit bat [4]. All viruses of the family *Paramyxoviridae* so far detected in bat species have been identified in fruit bats across Africa, Australia, South America, Asia, and Madagascar [3]–[6], [10]. Only a few studies attempting the isolation of paramyxoviruses in bats concerned insectivorous bat species, and all of them turned out with negative results [16], [17]. The only indication of paramyxoviruses in this group of bat species was the detection of Nipah virus antibodies in lesser Asiatic yellow bats (*Scotophilus kuhlii*) [16].

The present study aimed to detect and isolate novel paramyxoviruses in free-ranging European insectivorous bats and to estimate a possible impact of paramyxovirus infection on infected individual animals by comparing histo-pathological findings and virological results.

## Materials and Methods

As part of a study to investigate diseases in free-ranging bats in Germany [18], 120 deceased bats from 2009 of 15 different European vespertilionid species (*Eptesicus nilssoni, E. serotinus, Myotis bechsteini, M. daubentonii, M. mystacinus, M. nattereri, Nyctalus leisleri, N. noctula, Pipistrellus kuhli, P. nathusii, P. pipistrellus, P. pygmaeus, Plecotus auritas, P. austriacus, Vespertilio murinus*) were examined. The bat carcasses originated from 4 different geographic regions in Germany, i.e. Berlin greater metropolitan area (n = 83), Bavaria (n = 30), Brandenburg (n = 5), and Baden-Wuerttemberg (n = 2). Bat carcasses were stored at –20°C for transportation before performing a full necropsy. For histo-pathological examination, a small piece of tissue from all organs was fixed in buffered 4% formalin, processed routinely and embedded in liquid paraffin. Paraffin blocks were cut at 2–5 µm thickness and stained with hematoxylin-eosin [19]. Immuno-histochemistry was performed on all organs of PCR positive bats using rabbit immune sera against Beilong virus, J-virus, Menangle virus, Tioman virus and Nipah virus as described previously [20]. Samples of lung, liver, heart, and kidney, and conspicuous tissues (e.g. enlarged spleen) from each bat were homogenized in buffer and transferred to RNAlater (1∶1).

Pooled organ tissue from each bat was used for RNA/DNA extraction (PureLink^TM^ Viral RNA/DNA Mini Kit, Invitrogen, Germany) and further cDNA synthesis according to the manufacturer’s instructions (TaqMan® Reverse Transcription Reagents, Applied Biosystems, Germany). Broadly reactive paramyxovirus-specific RT-PCR assays were applied [21], yielding amplicons of 538 base pairs (PAR primers) and 486 base pairs (RES-MOR-HEN primers) located across domains I and II of the RNA polymerase (L)-coding sequence, a region of the genome suitable for phylogenetic analyses [22]. Since cDNA was readily available, PCR conditions were modified using the optimization method by Taguchi [23]. For this, based on the use of orthogonal arrays representing individual reactions with components at different concentration levels, a minimal number of experiments is allowed. To increase PCR sensitivity, the product yield for each reaction is used to calculate the optimal concentration of each reaction component. By using this method, a novel PCR reaction mixture was determined and henceforth used. For first-round PCR in the seminested assay, PCR mixtures contained 3 pmol each of forward and reverse primers, 1×Platinum® Taq buffer (Invitrogen), 250 nmol MgCl_2_ (Invitrogen), 2.5 pmol desoxynucleoside triphosphates (Invitrogen), 2 µl of cDNA, and 1.25 U of Platinum® Taq polymerase (Invitrogen). Water was then added to a final volume of 25 µl. The PCR mixture was sequentially incubated at 94°C for 2 min for denaturation, and then 40 cycles at 94°C for 15 s, 50°C for 30 s, 72°C for 30 s, and a final extension at 72°C for 7 min. For the second amplification in the seminested PCR assay, 1×Platinum® Taq buffer, 25 nmol MgCl_2_, 2.5 pmol desoxynucleoside triphosphates, 3 pmol each of forward and reverse primers, 1.25 U Platinum® Taq, 1 µl PCR product from the first reaction, adding water to a final volume of 25 µl. The cycling conditions were identical to the ones of the first round.

PCR products were run on an 1.5% agarose gel containing ethidium bromide. Images were captured on E.A.S.Y. RH-3 gel documentation system (Herolab, Germany). Amplicons from the PCR reaction were purified using the MSB® Spin PCRapace kit (Invitek, Germany). Both strands of the amplicons were sequenced with a BigDye Terminator v 3.1 Cycle Sequencing kit on an ABI Genetic Analyzer 3500 ×l D× automated sequencer (Applied Biosystems, Germany) using the corresponding PCR primers. Remaining reaction conditions were performed in accordance to the manufacturer’s protocol.

On the basis of newly acquired sequence information, specific qPCR assays were designed (Table 1) to screen pooled organ tissues of all 120 bats. Cycler conditions for all qPCR assays were as follows: predenaturation (95°C for 10 min), 45 amplification cycles (95°C for 30 s, 60°C for 30 s, 72°C for 30 s), and final extension (72°C for 10 min).

Additional primers were designed using conserved regions between Jeilongviruses and Henipaviruses to extend the sequence obtained by PAR primers (primers and protocol are available on request).

**Table 1:** Primer sequences.

Virus	Primer	Sequence 5′ to 3′	Tm (°C)
BatPV/Myo.mys/E20/09	E20/09 F	TgACAgATgATTTATgTgTTCgTTACT	55.6
	E20/09 R	gAATCCCACTCTgATTTCAACg	56.1
	E20/09 MGB	AAgTgTTTCATgCCATTgA	68
BatPV/Pip.pip/E95/09	E95/09 F	ggTgCTTggCCACCTCT	57.3
	E95/09 R	gCgATgAAgTTTgTCTTggA	56.4
	E95/09 MGB	CACTgCTTTATgCCTTTAA	70
BatPV/Nyc.noc/E155/09	E155/09 F2	ggAgATTgCACTCAgTCTTCCTgT	57.4
	E155/09 R2	gTCCCCCTACTTgAgATggCA	56.3
	E155/09 MGB	TCCgAgCTAAAATgTCA	68

Bayesian reconstruction of phylogenetic trees was performed in concordance with the current proposals of *Paramyxoviridae* taxonomy using *MrBayes*, version 3.1.2 [24], [25]. The underlying alignment by ClustalW was based on a 529 base pair fragment (PAR Primer) and 1,593 base pairs amplicon (long fragment) from PCR reactions. The evolutionary history was inferred using the bayesian MCMC method. First, a model selection for these calculations was performed with *jModelTest* [26] and model GTR+I+G (invariable sites, gamma distribution) was selected for the PAR and the RES-MOR-HEN fragment, and GTR+G to study the long fragment alignment. The calculation parameters were as follows: number of runs: four, number of generations: 1,000,000, sample frequency: 100, burn in: 25%. The results were finally visualized by the FigTree v1.2.1 program, a graphical viewer of phylogenetic trees. Based on the GTR substitution model the estimated transversion ratio, proportion of invariable sites and gamma distribution parameters were estimated automatically.

For confirmation of virus isolation and determination of the infected organs, RNA/DNA extraction and PCR analysis including sequencing was performed on all individual organs from infected bats. For two isolates, a second RT-PCR with primers RES-MOR-HEN [21] and the same PCR conditions as described above was conducted to acquire fragments comparable to previously isolated novel Henipa-like viruses from African fruit bats [4]. Bat species confirmation was achieved by sequencing and analyzing the mitochondrial DNA as described [27].

## Results

**Figure 1:**
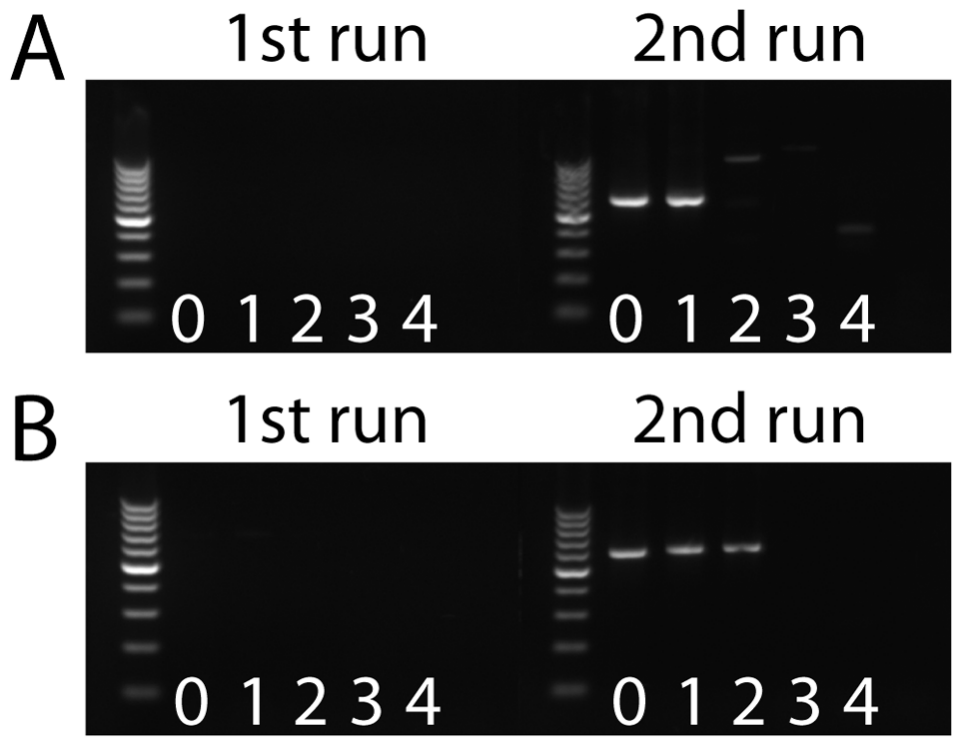
Improved detection sensitivity after Taguchi optimization of the *Paramyxovirinae* subfamily-specific PCR [**21**]. Gel electrophoresis of amplification products of the second round seminested PCR using 10-fold serial dilutions (10^0^ to 10^−4^) of a cDNA-sample (kidney of sample E20/09). (A) PCR protocol adopted for two-step PCR as previously published [21] using the pan-PAR-F1/PAR-R primer pair (1st run) and the pan-PAR-F2/PAR-R primer pair (2nd run). (B) Optimized protocol using the pan-PAR-F1/PAR-R primer pair (1st run) and the pan-PAR-F2/PAR-R primer pair (2nd run).

**Figure 2:**
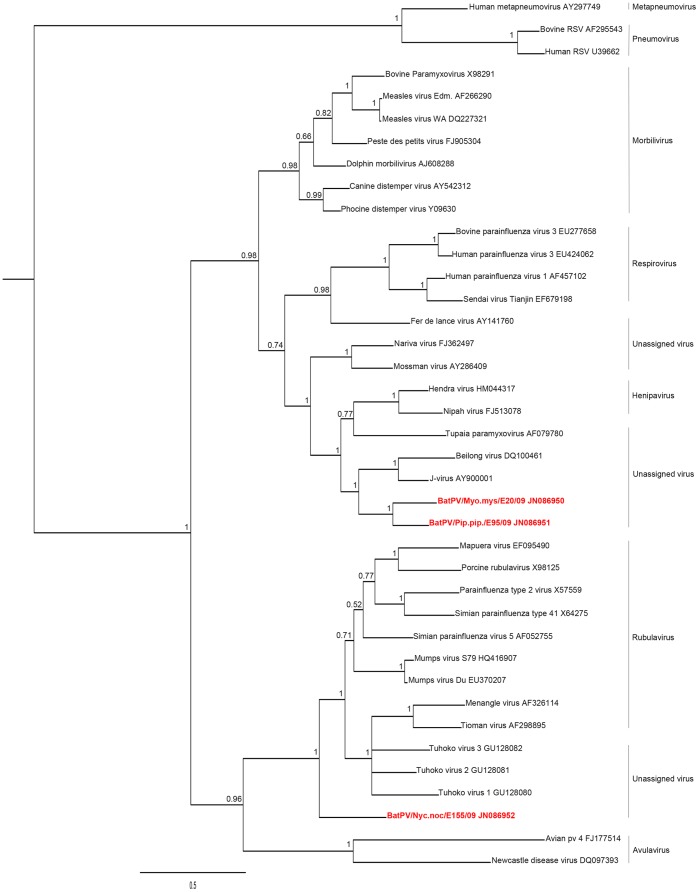
Phylogenetic analysis of the partial L-gene sequence obtained from PCR fragments after Pan-Paramyxovirinae-PCR with PAR primers (529 bp) [21]. The revealed gap-free alignment was used to generate a phylogenetic tree of the novel bat paramyxoviruses (red) concordant with representatives from all known genera of paramyxoviruses with MrBayes. Posterior probability rates are given next to the tree nodes. GenBank Accession numbers of novel paramyxoviruses PAR fragment: JN086950 (BatPV/Myo.mys/E20/09), JN086951 (BatPV/Pip.pip/E95/09), JN086952 (BatPV/Nyc.noc/E155/09). RSV  =  respiratory syncytial virus.

**Figure 3:**
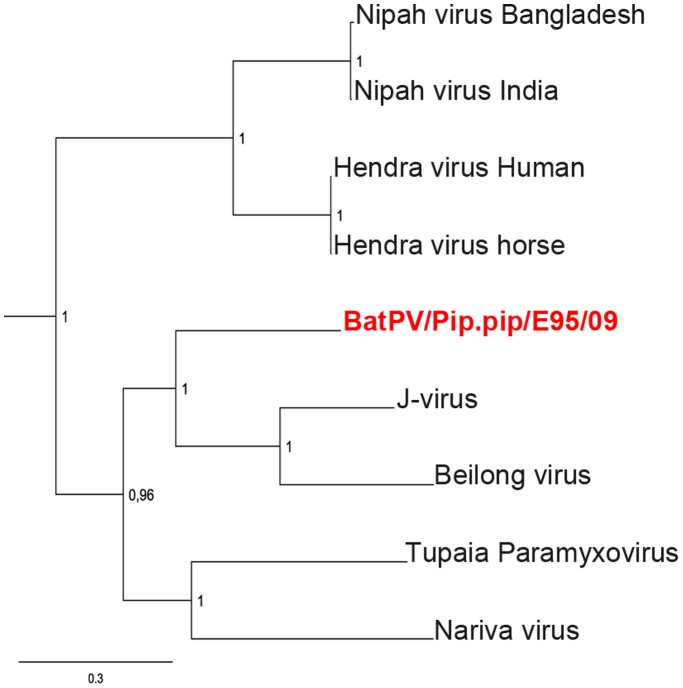
Phylogenetic analysis of the partial L-gene sequence obtained from a longer PCR fragment after Pan-Paramyxovirinae-PCR with novel primers (1,593 bp). The revealed gap-free alignment was used to generate a phylogenetic tree of the novel bat paramyxovirus (red) concordant with closely related representatives from other paramyxoviruses with MrBayes. Posterior probability rates are given next to the tree nodes. GenBank Accession number of the long fragment of BatPV/Pip.pip/E95/09: JN086951.

The modified PCR protocol (PAR primers) resulted in a 10-fold increase of sensitivity compared to the published protocol which was applied as a two-step PCR (Figure 1). With this optimization, three out of 120 pooled samples were PCR positive for paramyxoviruses using PAR primers (Table 2). The identified viruses were termed after the infected bat species: BatPV/Myo.mys/E20/09 (Accession number JN086950), BatPV/Pip.pip/E95/09 (Accession number JN086951), and BatPV/Nyc.noc/E155/09 (Accession number JN086952). Fragments of 529 bp length were aligned with homologous fragments of the partial polymerase gene of other members of the family *Paramyxoviridae* from GenBank (Figure 2). Phylogenetic analysis confirmed three distinct isolates within the subfamily *Paramyxovirinae*. BatPV/Nyc.noc/E155/09 was in basal association to other members of the genus Rubulavirus. For both BatPV/Myo.mys/E20/09 and BatPV/Pip.pip/E95/09, the closest association was observed to J-virus and Beilong virus (unclassified viruses) [28], [29]. A longer sequence of 1,593 bp was generated for BatPV/Pip.pip/E95/09 and used for an extended phylogenetic analysis (Figure 3). The highest similarity was revealed for BatPV/Myo.mys/E20/09 to J-Virus with 66.4%, for BatPV/Pip.pip/E95/09 also to J-Virus with 64.1%, and for BatPV/Nyc.noc/E155/09 to Rubulavirus with 62.1% (Table 3). Within the subfamily *Paramyxovirinae* the extent of minimal nucleotide homology for the partial polymerase gene between different viruses in the same genus ranges from 64.1% (Rubulavirus) to 76.8% (Henipavirus), whereas the extent of nucleotide similarity between viruses from different genera is between 40.3% (Morbillivirus) and 58.1% (Henipavirus). Analysing the nucleotide homology of the partial polymerase gene of the new insectivorous bat paramyxoviruses, no definite correlation to one of the other paramyxovirus genera could be obtained. The comparison of sequences of BatPV/Myo.mys/E20/09 (Accession number JN086953) and BatPV/Pip.pip/E95/09 (Accession number JN086954), obtained from the PCR assay with RES-MOR-HEN primers (Table 2), confirmed the results of the above-mentioned phylogenetic analysis (data not shown).

**Table 2:** Animal properties of paramyxovirus-infected adult insectivorous bats.

Virus	Species	Sex	Source	Positive PCR/sequencing (PAR primers)		Positive PCR/sequencing (RES-MOR-HEN primers)	
				Organ pool	Organ	Organ pool	Organ
BatPV/Myo.mys/E20/09	*Myotis mystacinus*	Male	Bavaria	+	Kidney	+	+
BatPV/Pip.pip/E95/09	*Pipistrellus pipistrellus*	Female	Bavaria	+	n.d.*	+	n.d.*
BatPV/Nyc.noc/E155/09	*Nyctalus noctula*	Female	Berlin	+	Lung	­–	−

* not determined due to sample volume limitations.

**Table 3:** Relatedness of the novel paramyxoviruses to other members of currently established genera of the family *Paramyxoviridae*.

Genus / Species	(1)	(2)	(3)	(4)	(5)	(6)	(7)	(8)	(9)	(10)	(11)
(1) Pneumovirus	n.d.										
(2) Metapneumovirus	63.8	n.d.									
(3) Avulavirus	31.3	32.7	n.d.								
(4) Rubulavirus	33.8	34.2	48.8	≥64.1							
(5) Respirovirus	33.4	32.9	44.2	46.0	≥73.0						
(6) Morbillivirus	34.4	33.5	42.7	45.9	52.5	≥74.2					
(7) Henipavirus	34.2	33.2	37.1	39.5	44.0	45.7	≥76.8				
(8) J-virus	34.7	35.3	42.5	48.0	54.0	58.8	48.6	n.d.			
(9) BatPV/Myo.mys/E20/09*	37.5	37.5	38.8	46.8	54.0	58.9	63.2	**66.4**	n.d.		
(10) BatPV/Pip.pip/E95/09	36.7	36.9	45.2	50.0	53.0	58.0	51.1	**64.1**	**74.5**	n.d.	
(11) BatPV/Nyc.noc/E155/09*	36.8	36.1	46.2	**62.1**	47.4	49.1	47.2	48.5	47.5	47.9	n.d.

Table lists percentage of nucleotide homology within the partial L-gene (529 base pairs) obtained by pan-*Paramyxovirinae*-specific PCR (PAR primers). Bold numbers present the highest identities of the novel bat paramyxoviruses with the following established species: Human respiratory syncytial virus M74568 (Pneumovirus), Human metapneumovirus AY297749 (Metapneumovirus), Newcastle disease virus AY505072 (Avulavirus), Mumps virus HQ416907 (Rubulavirus), Sendai virus EF679198 (Respirovirus), Measles virus AF266290 (Morbillivirus), Hendra virus HM044317 (Henipavirus), J-virus (AY900001), BatPV/Myo.mys/E20/09 (JN086950), BatPV/Pip.pip/E95/09 (JN086951), BatPV/Nyc.noc/E155/09 (JN086952). n.d.: not determined.

The first paramyxovirus termed BatPV/Myo.mys/E20/09 was detected in pooled organs and was subsequently confirmed in the kidney only of one adult male whiskered bat (*Myotis mystacinus*) found in Bavaria. Histological examination of the internal organs revealed multifocal mild interstitial nephritis with lymphoplasmacytic infiltrates and occasional neutrophiles. Lungs had mild non-suppurative interstitial pneumonia and marked leucocytostasis in most blood vessels. Additionally, there was distinct activation of the lymphoreticular tissue of the spleen with moderate follicular hyperplasia and sparse irregularly distributed small foci of lymphocytes and plasma cell aggregations within the liver.

**Figure 4:**
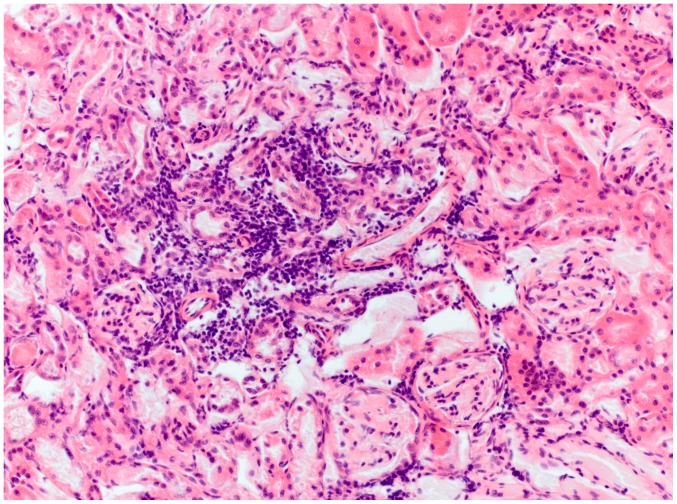
Kidney of a common pipistrelle bat (E95/09) with interstitial nephritis comprised by segmental infiltrates of lymphocytes, plasma cells, and occasional single neutrophilic granulocytes.

The second virus (BatPV/Pip.pip/E95/09) was detected in the pooled organs of an adult female common pipistrelle bat (*Pipistrellus pipistrellus*) also found in Bavaria. No specific infected organ could subsequently be determined due to sample size limitations. Histologically the animal had multifocal moderate interstitial nephritis with segmental infiltrates of lymphocytes, plasma cells, and occasional single neutrophiles (Figure 4). There was mild generalized interstitial pneumonia and moderate follicular hyperplasia of the spleen. There were mild intrasinusoidal infiltrates of neutrophiles, lymphocytes, and plasma cells within the liver.

The third virus (BatPV/Nyc.noc/E155/09) was detected in pooled organs and was subsequently confirmed in the lung of only one adult female noctule bat (*Nyctalus noctula*) found in Berlin. Histologically the bat revealed marked follicular hyperplasia of the spleen without further inflammatory organ lesions. The lung was severely congested, and oedematous fluid was present in the lung parenchyma.

Using immune sera against Beilong virus, J-virus, Menangle virus, Tioman virus and Nipah virus in immuno-histochemistry, no stained antigens were visualized in any of the paramyxovirus-positive bats although all immune sera worked well against their homologous virus in corresponding positive controls.

After screening all 120 bats of 15 species with three virus-specific qPCR assays, an identical paramyxovirus to BatPV/Myo.mys/E20/09 was detected in the spleen of one additional *Myotis mystacinus* (E120/09).

## Discussion

During the past decade, bats have increasingly been recognized as members of the animal group with the highest relative risk to harbour novel emerging zoonotic pathogens [2]. The emergence of Hendra and Nipah virus provided the first evidence of a zoonotic paramyxovirus originating from bats with a broad host range including humans. Interestingly, despite the enormously diverse chiropteran animal order, so far, with the exception of rabies, only fruit bats have been implicated as a reservoir of a number of new and emerging zoonotic viruses [3]–[10].

With this study, we were able to describe the detection and characterization of the first three paramyxoviruses in insectivorous bats. The genetic distance between these three novel paramyxoviruses and the closest related member known is higher than that of members within other paramyxovirus genera, suggesting that all three viruses might be considered as unassigned paramyxoviruses. Thus the two viruses BatPV/Myo.mys/E20/09 and BatPV/Pip.pip/E95/09 might even be considered as members of a new putative genus, as they contain an amino acid identity of 79.5% of the partial L-gene, the highest conserved region of the paramyxovirus genome. Further precise genetic analyses will have to prove whether they ought to be integrated into the proposed new genus Jeilongvirus [29], comprising J-virus and Beilong virus, with amino acid identities between 69.9% and 74%, respectively, although their viral antigens in PCR positive organs are not immunologically cross-reactive. The third novel paramyxovirus, BatPV/Nyc.noc/E155/09, has a basal association with the genus Rubulavirus and could therefore become a member of this genus, although nucleic acid identity was slightly lower than that between already classified members within this genus and no cross-reactivity of viral antigens with immune sera of closely related rubulaviruses was obtained.

Besides virus detection, our study allowed a direct correlation of virology and histo-pathology results. In previous studies in which bats were examined for paramyxovirus infections, no overt clinical disease was noted [5], [30], despite occasional high prevalences of antibodies against various paramyxoviruses (e.g. Hendra and Nipah virus) and the detection of paramyxovirus RNA in different bat organs [5], [30]–[32]. For Hendra virus infections of pteropid bats, the subclinical course has been confirmed by experimental infection. Kidneys are the only site of pathological lesions after Hendra virus infection of pteropid bats, with mild interstitial perivascular infiltrates by mononuclear inflammatory cells, while virus nucleic acids were also detected in lung, spleen, gastrointestinal tract, and urine [33]. In contrast, Nipah virus was only detected in kidneys (male) and uteri (female) of pteropid bats after experimental infection [34]. In all Hendra virus and Nipah virus experimental infection studies the amount of virus recovered reached the limit of detection level, a similar situation encountered in our study. In infected insectivorous bats, low band intensities of PCR products of organs tested positive for paramyxovirus infection indicated a low virus load. Likewise, the kidneys were the only organ infected in the male whiskered bat (BatPV/Myo.mys/E20/09) and presumably in the female common pipistrelle. Interestingly, both animals had mononuclear inflammatory interstitial infiltrates similar to the reported experimental Hendra virus infections. Unfortunately, attempts to prove the evidence by specific immunohistochemistry with antibodies directed against Beilong virus, J-virus, Menangle virus, Tioman virus and Nipah virus were not successful. A number of reasons could account for this result. Either the noted inflammatory changes were indeed unrelated to paramyxovirus infection or the immunogenic epitopes of paramyxoviruses in European insectivorous bats differ significantly from their australo-asian relatives, hence prohibiting bonding between the reagents or, taking into account that molecular investigations indicated a low virus load, the number of infectious particles was too low to be picked up by immunohistochemistry. Although unequivocal evidence of the causative association between paramyxovirus infection and renal inflammation *remains open* it is important to note, that nephritis is a rare finding in insectivorous bats. Out of 500 examined deceased bats only 3% had inflammatory changes within their kidneys, while 20% of these respective cases were clearly associated to bacterial disease [19]. Considering this background information together with findings from experimental paramyovirus infections in fruit bats a possible link between the renal mononuclear infiltrates and the detected novel paramyxovirus nucleic acids seems feasible. With this, a possible transmission route via urine like in Hendra virus infection could also be assumed for these viruses. In contrast, the detection of BatPV/Nyc.noc/E155/09 limited to the lungs of the female noctule bat is suggestive of an oronasal and/or salivary route of transmission. Our findings confirm a promising approach for the ultra-sensitive detection of paramyxoviruses by applying a modified PCR protocol as a powerful tool, including non-invasive sampling (oral swabs, urine, faeces). However, it should be emphasized that substantial insights regarding the estimation of possible spill-over events can only be achived by a combination of virology, histo-pathology, and bat ecology investigations.

Emerging paramyxoviruses from fruit bats in spill-over hosts have regularly been associated with ecosystem and land-use changes resulting in an increased overlap of bats, domestic animals, and human ecologies and thereby increased opportunities for bat-borne zoonotic diseases to emerge [35]. As demonstrated in this study, paramyxoviruses basally related to Henipaviruses also exist in geographic areas distant to the distribution range of fruit bats, the suspected natural hosts for Henipaviruses, indicating a possible virus–host co-evolution beyond this animal group. Since infected bats were found in close proximity to heavily populated human habitations as well as intensive agricultural use, a potential risk for the emergence of zoonotic paramyxoviruses in Europe should be further elucidated. Although the three novel paramyxoviruses detected in three distinct European bat species cannot be readily assigned to any previously described paramyxovirus genus, they are associated to two very distinct genera (Rubulavirus and Jeilongvirus) [22], indicating a similarly broad genetic diversity among paramyxoviruses in insectivorous bats compared to fruit bats. Given the much larger diversity amongst insectivorous bats with over 1,000 bat species in contrast to 186 fruit bat species [36], we predict a far higher diversity of paramyxoviruses in insectivorous bats. Extensive phylogenetic studies of insectivorous bat-born paramyxoviruses will provide further insight into the suggested co-evolution of paramyxoviruses and bats [35]. In addition to Africa, Australia, South America, and Asia, the detection of novel paramyxoviruses in European bats extends the possible geographic overlap with other susceptible spill-over hosts.

Is there a possibility for paramyxoviruses of insectivorous bats to emerge as zoonotic pathogens? Before any answer to this question can be attempted, further research on paramyxovirus diversity and distribution combined with the understanding of dynamics of pathogen cycles within bat populations will be needed as well as investigations into pathogenicity factors of these viruses, like receptors for host invasion. Particularly as so far transmission of bat related paramyxoviruses did not occur directly between bats and humans but depended on a secondary host species like horses or pigs.
